# Supplementary material for: Prevalence and Awareness of Medication Overuse Headache among Undergraduate Students at the University of Belgrade
Source: Brain Sci. 2024 Sep 19;14(9):938. doi: 10.3390/brainsci14090938 (PMC11429653; doi:10.3390/brainsci14090938)
Supplement: Supplementary file 1 [file brainsci-14-00938-s001.zip › brainsci-3108198-File S1.pdf]

Assessment of the prevalence and awareness of Medication Overuse Headache in the Undergraduate Student Population

Dear colleagues, we are Ana Milicevic and Dajana Orlovic, students of the Faculty of Medicine, University of Belgrade conducting research entitled "Assessment of the prevalence and awareness of Medication Overuse Headache in the Student Population at the University of Belgrade".

We present to you an anonymous questionnaire that has 17 questions and takes approximately 4-5 minutes to complete. Participation in this research is entirely voluntary; you can choose whether or not you want to participate. If you accept, your participation will significantly enhance the progress of our project.

This research is approved by the Ethical committee of the Faculty of Medicine, University of Belgrade. The information collected during the research will be kept confidential.

Thank you for your time.

1. Do you agree to participate in this research?
   A) Yes, I agree
   B) No, I don't agree

2. Gender:
   A) Male
   B) Female

3. Age:
   A) 21 years old or younger
   B) Between 22 and 25 years old
   C) 25 years old or older

4. I'm currently an undergraduate student studying at:
   A) School of Medicine, University of Belgrade
   B) Other schools at the University of Belgrade
   C) I am not an undergraduate student at the University of Belgrade

5. Did you use any of the following painkillers in the last 3 months? (mark all the answers that apply)
   A) Nonsteroid analgetics (Aspirin, Andol, Midol, Ibuprofen, Brufen, Rapidol, Spedifen, Caffetin Menstrual, Diclofenac, Rapten K; Nalgesin…)
   B) Paracetamol (Panadol, Febricet)

C) Combined analgetics (Caffetin, Caffebol, Panadol Extra, Fervex, Coldrex, Efferalgan, Metafex, Tylol Hot…)
D) Triptans and/or ergotamines (Sumatriptan, Imigran, Trecar, Amigren)
E) I do not use painkillers

6. Why do you use painkillers?
   A) Mainly for headaches
   B) Mainly for other types of pain
   C) I do not use painkillers

7. Please state all the side effects of painkillers that you are aware of: (mark all the answers that apply)
   A) Loss of appetite
   B) Abdominal pain
   C) Nausea, vomiting, diarrhea
   D) Chronic headache
   E) Fatigue
   F) Skin reactions
   G) Liver damage
   H) Kidney damage
   I) I am not familiar with the side effects of these medications

8. In the past year, have you experienced a headache that was not related to a cold, hangover, or head injury?
   A) Yes
   B) No

9. Have you ever consulted a doctor for a headache?
   A) Yes
   B) No

10. How many days in the last 3 months have you experienced a headache?
    A) Rarely (average once a month or less)
    B) 2-9 days per month
    C) 10-14 days per month
    D) 15 days or more per month
    E) I do not have headaches

11. Which best describes your most bothersome headache?
    A) Pressing, squeezing, or tightening pain

B) Throbbing or pulsating pain
C) I do not have headaches

12. Does your most bothersome headache usually affect one or both sides of your head?
    A) One side
    B) Both sides
    C) I do not have headaches

13. During this type of headache, do you experience any of the following symptoms: (mark all the answers that apply)
    A) Nausea (as though you may vomit or throw up)
    B) Vomiting
    C) Daylight or other lighting bothers you (you prefer to be in the dark)
    D) Noise bothers you (you prefer to be in the quiet)
    E) Your headache tends to worsen during exercise (like walking or climbing stairs)
    F) I only have headaches, without any other symptoms
    G) I do not have headaches

14. How intense is this type of headache usually?
    A) Mild
    B) Moderately strong
    C) Severe
    D) I do not have headaches

15. Without taking any painkillers (or if treated unsuccessfully) how long does this type of headache usually last?
    A) _________ hours
    B) I do not have headaches

16. How often have you taken painkillers in the past 3 months?
    A) Rarely (on average once a month or less)
    B) On average 2-14 days per month
    C) More than 14 days per month
    D) I have not taken painkillers

17. If you knew that regular use of painkillers could cause frequent headaches, would you change anything?
    A) Nothing
    B) I would reduce or stop taking painkillers
    C) I would consult a doctor
